# Effect of Two Unique Nanoparticle Formulations on the Efficacy of a Broadly Protective Vaccine Against *Pseudomonas Aeruginosa*

**DOI:** 10.3389/fphar.2021.706157

**Published:** 2021-08-18

**Authors:** Debaki R. Howlader, Sayan Das, Ti Lu, Gang Hu, David J. Varisco, Zackary K. Dietz, Sierra P. Walton, Siva Sai Kumar Ratnakaram, Francesca M. Gardner, Robert K. Ernst, William D. Picking, Wendy L. Picking

**Affiliations:** ^1^ Department of Pharmaceutical Chemistry, University of Kansas, Lawrence, KS, United States; ^2^ Department of Microbial Pathogenesis, University of Maryland, Baltimore, MD, United States

**Keywords:** pseudomonas, T3SS vaccine, L-PaF, nanoparticle vaccine, protein formulations, opsonophagocytosis, IL-17

## Abstract

*Pseudomonas aeruginosa* is an opportunistic pathogen responsible for a wide range of infections in humans. In addition to its innate antibiotic resistance, *P. aeruginosa* is very effective in acquiring resistance resulting in the emergence of multi-drug resistance strains and a licensed vaccine is not yet available. We have previously demonstrated the protective efficacy of a novel antigen PaF (Pa Fusion), a fusion of the type III secretion system (T3SS) needle tip protein, PcrV, and the first of two translocator proteins, PopB. PaF was modified to provide a self-adjuvanting activity by fusing the A1 subunit of the heat-labile enterotoxin from Enterotoxigenic *E. coli* to its N-terminus to give L-PaF. In addition to providing protection against 04 and 06 serotypes of *P. aeruginosa*, L-PaF elicited opsonophagocytic killing and stimulated IL-17A secretion, which have been predicted to be required for a successful vaccine. While monomeric recombinant subunit vaccines can be protective in mice, this protection often does not transfer to humans where multimeric formulations perform better. Here, we use two unique formulations, an oil-in-water (o/w) emulsion and a chitosan particle, as well as the addition of a unique TLR4 agonist, BECC438 (a detoxified lipid A analogue designated Bacterial Enzymatic Combinatorial Chemistry 438), as an initial step in optimizing L-PaF for use in humans. The o/w emulsion together with BECC438 provided the best protective efficacy, which correlated with high levels of opsonophagocytic killing and IL-17A secretion, thereby reducing the lung burden among all the vaccinated groups tested.

## Introduction

*Pseudomonas aeruginosa* (Pa) is an important opportunistic human pathogen responsible for severe infections in patients with burns, severe wounds, pneumonia, and critically ill patients who require intubation (ventilator-associated pneumonia) or catheterization (urinary tract infections) (Grimwood et al., 2015; Anantharajah et al., 2016). Clearing Pa has become increasingly difficult due to innate and acquired antibiotic resistance (Anantharajah et al., 2016). Multidrug-resistant (MDR) Pa was classified as a serious threat in the CDC Antibiotic Resistance Threats report 2019 (CDC, 2019). In 2017, there were ∼32,600 cases of MDR Pa infection in hospitalized patients causing an estimated 2,700 deaths and costing $757 million in health care costs in the United States (CDC, 2019). A 2016 report describes Pa as the most common Gram-negative infection amongst troops with combat-related injuries in Afghanistan with 10% being MDR (Merakou et al., 2018). Pa is also the major cause of pulmonary infections in cystic fibrosis (CF) patients with >70% of this group being chronically colonized by their late teens (Grimwood et al., 2015). Pa infections in chronic pulmonary conditions such as chronic obstructive pulmonary disease (COPD) and non-CF bronchiectasis (nCFB) have poor prognoses (Loebinger et al., 2009; Almagro et al., 2012). Despite the ability to cause disease in humans who have been injured or hospitalized, aging represents the biggest risk factor for acquiring acute lethal Pa infection (Merakou et al., 2018).

There are no licensed vaccines to prevent Pa infections, but several are in the pipeline (Merakou et al., 2018; Baker et al., 2020). Many of the vaccines, however, only protect against a subset of Pa strains. In contrast, the proteins of the surface localized type three secretion system apparatus (T3SA) are highly conserved. PcrV and PopB are essential surface localized T3SS proteins that are >96–99% conserved among PAO1/PA14-like strains (Winsor et al., 2016). Because these are T3SS scaffold proteins required for the early stages of infection for these strains (Goure et al., 2004), vaccine escape is reduced due to the fact that mutations in these proteins would impact assembly of an active T3SS and render them nonpathogenic (Goure et al., 2004). When delivered intranasally (IN), we demonstrated that PcrV + PopB admixed with dmLT (double-mutant labile toxin from Enterotoxigenic *E. coli*) protected mice against acute Pa pulmonary challenge (Das et al., 2020). To reduce costs associated with their production and formulation, we genetically fused PcrV and PopB to produce PaF (Pa Fusion), which elicited protection against Pa in mouse and rat models (Das et al., 2020). To further reduce the potential reactogenicity associated with IN delivery of dmLT (Norton et al., 2012), we fused the A1 subunit (LTA1) to PaF (L-PaF) (Das et al., 2020). LTA1 has been shown to stimulate a balanced Th1/Th2/Th17 and mucosal immune response characterized by production of IgA and IL-17A (Norton et al., 2012). When delivered IN to mice and rats, L-PaF elicited strong IgG and IgA titers with high levels of opsonophagocytic killing (OPK) activity. It also expedited the serotype independent clearance of homologous and heterologous Pa strains from the lungs of the challenged rodents (Das et al., 2020). It has been postulated that high levels of OPK and IL-17A are important for generating a protective immune response in humans against Pa (Merakou et al., 2018; Sainz-Mejias et al., 2020).

Vaccination is perhaps the greatest public health achievement of our time and L-PaF represents a unique subunit vaccine platform for preventing Pa infections. Nevertheless, while protective antigens have been identified and shown to be successful as vaccines in mice, they often fail once they are introduced into human trials (Marasini et al., 2017; Corthesy and Bioley, 2018). Some of these failures are associated with the use of soluble antigens with adjuvants that can elicit a significant immune response in rodents, but do not elicit the same response in humans (Marasini et al., 2017; Corthesy and Bioley, 2018). In addition to preclinical formulation to develop a stable protein formulation, studies have shown a better response in humans when the antigen is presented as a multimer in the context of a nanoparticle (Marasini et al., 2017; Corthesy and Bioley, 2018). Many nanoparticle formulations are now being tested for use in intramuscular and intranasal routes. The most well-known multimerization method is the use of aluminum salts such as Alhydrogel, however, the adjuvant activity of aluminum salts tends to skew the immune response to a Th2 response, which is more aligned with the humoral response and not the balanced responses often required for clearing mucosal pathogens (Gregg et al., 2018).

Herein, as a method to create multimers of L-PaF, we formulate L-PaF into two unique formulations with respect to size, composition and mechanism of immune system induction. We show that an o/w emulsion, is superior to the use chitosan nanoparticles. This o/w emulsion contains squalene, which has been shown to promote protection against influenza in an older population (Wagner and Weinberger, 2020) Additionally, we have added the Bacterial Enzymatic Combinatorial Chemistry candidate 438 (hereafter referred to as BECC438), a novel TRL-4 agonist that is a bisphosphorylated and detoxified lipid A biosimilar (Gregg et al., 2018). In addition to fusion with LTA1, use of BECC438 further promotes a balanced Th1-Th2 immune response and increases protection elicited by PaF and further decreases the antigen dose needed to elicit protection.

## Materials and Methods

### Materials

Squalene was purchased from Echelon Biosciences (Salt Lake City, UT), Chitosan and C48/80 from Millipore-Sigma (St. Louis, MO). All other buffers chemicals were reagent grade.

### Protein Preparation

L-PaF was made as previously described (Das et al., 2020). Briefly, *E. coli* Tuner (Goure, Pastor et al.) cells expressing L-PaF/His-Tag PcrH were grown in TB media supplemented with chloramphenicol (34 μg/ml) with a fed-batch mode in a 10 L bioreactor (Labfors 5, Infors USA Inc., MD). An overnight starter was expanded to 1L and ∼800 ml was transferred to the bioreactor containing 9 L of TB media supplemented with chloramphenicol (34 μg/ml). The culture temperature was maintained at 30°C and protein expression was induced adding IPTG to 1 mM when the culture reached an A_600_ of ∼25. After 3 h, the bacteria were collected and processed for purification. The L-PaF/His-Tag PcrH was captured on an IMAC column followed by Q anion exchange chromatography. Lauryldimethylamine oxide (LDAO) was added to a final concentration of 0.1% to release the HT-PcrH. The protein solution was passed over a final IMAC column with the L-PaF passing through the column. L-PaF was dialyzed into PBS with 0.05% LDAO and stored at −80°C. LPS levels were determined using a NexGen PTS with EndoSafe cartridges (Charles River Laboratories, Wilmington, MA). All proteins had LPS levels <5 Endotoxin units/mg protein based on analysis using an Endosafe system (Charles River Labs). Each of the proteins used here (PcrV, PopB, PaF and L-PaF) were checked for quality and purity using SDS-PAGE (Supplementary Figure S1) [(Das et al., 2020)].

### Preparation of L-PaF ME and L-PaF BECC438/ME Formulations

Squalene (8% by weight) and polysorbate 80 (2% by weight) were mixed to achieve a homogenous oil phase. Using a Silverson L5M-A standard high-speed mixer, 40 mM Histidine (pH 6) and 20% sucrose were added to the oil phase and mixed at 7,500 RPM followed by six passes in a Microfluidics 110P microfluidizer at 20,000 psi to generate a milky emulsion of 4XME (MedImmune Emulsion). Polysorbate 80 acted as an emulsifying agent to stabilize the emulsion. BECC438 (2 mg/ml) was prepared in 0.5% triethanolamine by vortexing followed by sonicating for 30 min in a 60°C water bath sonicator until the BECC438 was completely dissolved. The pH of BECC438 solution was adjusted to 7.2 with 1 M HCl. To make the L-PaF with ME, the protein was added to the ME with a final concentration of 0.67 mg/ml, vortexed and allowed to incubate overnight at 4°C. To make the L-PaF with ME and BECC438 formulation, ME and BECC438 were mixed by vortexing for 2 min and incubated overnight at 4°C. The next day, L-PaF was mixed with ME-BECC438 solution at a volumetric ratio of 1:1 to achieve desired final antigen concentration.

### Preparation of L-PaF Chitosan-C48/80 (Chi) and L-PaF BECC438/Chi Formulations

To make chitosan nanoparticles, 1 gm of chitosan was added in 10 ml of a 1 M NaOH solution and stirred for 3 h at 50°C (Gan and Wang, 2007). The chitosan solution was then filtered through 0.45 µm membrane and the resulting pellet was washed with 20 ml of MilliQ water. The recovered chitosan was resuspended in 200 ml of 1% (v/v) acetic acid solution and stirred for 1 h. The solution was filtered through 0.45 µm membrane, and 1 M NaOH was added to adjust the pH to 8.0, resulting in purified chitosan. Purified chitosan was vacuum dried for 24 h at 40°C. C48/80 loaded chitosan nanoparticles (Chi) were prepared by adding dropwise 3°ml of an alkaline solution (5 mM NaOH) containing C48/80 and Na_2_SO_4_ (0.3 mg/ml and 2.03 mg/ml, respectively) to 3 ml of a chitosan solution (1 mg/ml in acetic acid 0.1%) with high-speed vortexing. The Chi was formed using magnetic stirring for an additional 1 h (Bento et al., 2015). Chi was then collected by centrifugation at 4500Xg for 30 min and the pellet resuspended in MOPS buffer (20 mM, pH 7). The L-PaF in PBS was exchanged into MOPS buffer (20 mM, pH 7) using an Amicon Ultra-4 centrifugal filter. To make L-PaF Chi, L-PaF was added to the Chi solution to a weight ratio of 1:4. To make L-PaF BECC438/Chi, the nanoparticles were mixed with BECC438 by vortexing and incubating for 10 min. L-PaF was then added, mixed by vortexing and incubated for 2 h at 4°C.

### Intrinsic Tryptophan Fluorescence

Intrinsic tryptophan fluorescence spectra were obtained as described previously (Wei et al., 2018a; Wei et al., 2018b). Briefly, intrinsic tryptophan fluorescence was measured by a fluorescence plate-reader (Fluorescence Innovations, Minneapolis, MN), which is equipped with a tunable pulsed dye laser, a temperature controlled 384-well sample holder (Torrey Pines Scientific, Carlsbad, CA), and a high-speed digitizer. L-PaF and formulated L-PaF samples (20 µl) were loaded into a Hard-Shell 384-well PCR plates. Samples were excited at 295 nm and steady state emission spectra were collected using a charged coupled device detector from 310 to 400 nm. Fluorescence moment (mean center of spectra mass peak position or MSM peak position) was reported. Temperature ramps were set from 10 to 95°C with an increment of 1°C per step and an equilibration time of 60 s at each temperature. Moment (MSM peak position) were plotted as a function of temperature and first derivative of the resulting data was used to calculate the melting temperature (T_m_) using Origin 7.0 (OriginLab, Northampton, MA).

### Size and Zeta Potential

The hydrodynamic diameter of L-PaF and formulations were determined using dynamic light scattering (DLS) with Zetasizer Ultra (Malvern Instruments). Formulations were diluted in 1:10 with water in triplicate and measured in disposable polystyrene cuvettes. The SOP parameters were set up as following: material RI = 1.59, dispersant RI (water) = 1.33, T = 25°C, viscosity (water) = 0.887 cP, measurement angle = 173° backscatter with automatic attenuation. The Z-average values of the hydrodynamic diameter of samples were calculated via cumulant analysis. To gain more information on the particle size and concentration, Multi-Angle Dynamic Light Scattering (MADLS) measurements with the Zetasizer Ultra were performed to collect the intensity of backscattering, forward scattering, and side scattering.

Zeta potential measurements were performed via electrophoretic light scattering using the Zetasizer Ultra. Samples were diluted 10-fold in water before analysis. Samples were introduced into disposable folded capillary cells at 25°C. Scans were performed until the results had acceptable correlation functions (typically 50–100 scans). Three independent measurements were performed, and the zeta potential was calculated based on electrophoretic mobility of sample particles.

### Mice and Immunizations

The mouse animal protocols were reviewed and approved by the University of Kansas Institutional Animal Care and Use Committee Practices (protocol AUS 222-03). Six-to eight-week-old female BALB/c mice (n = 10/group) (Charles River Laboratories, Wilmington, MA) were used for all experiments. Prior to administration, the following were prepared in 30 µl volumes: PBS, 1 μg L-PaF (L-PaF), 1 μg L-PaF in ME (L-PaF ME), 1 μg L-PaF in BECC438-ME (L-PaF BECC438/ME), 1 μg L-PaF in Chitosan-C48/80 (L-PaF Chi), and 1 μg L-PaF in BECC438-Chitosan-C48/80 (L-PaF BECC438/Chi). For immunizations, mice were anesthetized using isoflurane and vaccine formulations administered intranasally (IN) as previously described (Martinez-Becerra et al., 2012). Immunizations were on days 0, 14, and 28 for this study. Blood was collected prior to each vaccination and at days 42 and 56.

### Antigen-Specific IgG and IgA

Antibodies specific for PcrV and PopB were determined by ELISA, as described previously (Das et al., 2020). Briefly, 96-well plates coated with PcrV or PopB (1 μg/ml in PBS) were blocked overnight with 10% milk (Omniblok, americanbio) in PBS. Each well was incubated with serum samples for 1 h at 37°C. After washing the plates with PBS-Tween (0.05%), secondary antibody (KPL, Gaithesburg, MD) was added and incubated for 1 h at 37°C. Levels of IgG (H + L) and IgA were determined using horseradish peroxidase-conjugated secondary antibodies (human serum adsorbed) raised in goat (Southern Biotech, Birmingham, AL). 3,3′,5,5′-Tetramethylbenzidine (TMB) substrate was added, and reaction was stopped with H_3_PO_4_. Endpoint titers were calculated and represented as ELISA units per mL (EU ml^−1^).

### Opsonophagocytosis Assay

OPK was carried out as previously described (Das et al., 2020). Briefly, Pa strain mPA08-31 was grown overnight at 37°C. A new culture was started by adding 200 µl of the overnight culture to 20 ml of LB media and grown to A_600_ of 0.3. Bacteria were collected by centrifugation and a portion of the resuspension adjusted to a concentration of 2x10^7^ cells/mL in Minimal Essential Medium (MEM, ThermoFisher, Waltham, MA) containing 10% bovine serum albumin (BSA, Sigma, St. Louis, MO). The J774A.1 (ATCC, Manassas, VA) murine macrophage cell line was grown to 90% confluency in Dulbecco’s Modified Eagle’s Medium (DMEM, ThermoFisher, Waltham, MA) and was adjusted to allow for a final multiplicity of infection (MOI) of 0.1 in 10% MEM-BSA. At 42 Days Post-Immunization (DPIm) sera from the vaccinated mouse groups (5 µl from each mouse were mixed) were heat treated at 56°C for 30 min to destroy complement. Serum (1:500), bacteria and macrophages were mixed at a 1:1:1 ratio to a final volume of 300 µl and kept for 30 min at 37°C. The suspension was then serially diluted and plated on *Pseudomonas* Isolation Agar (PIA, BD, Franklin Lakes, NJ). Percent killing = {[(CFU from T_0_)–(CFU from T_30min_)]/(CFU fromT_0_) × 100}. An appropriate serum control was used where MEM-BSA was used in the published protocol. Technical quadruplets from each group were assessed and statistical comparisons made as described below.

### IL-17A ELISpot

Immunized mouse lungs were extracted and processed to single cell suspensions according to manufactures specifications (Miltenyibiotec). Lung cells (1 x 10^6^ cells/well) were incubated for 24 h at 37°C in the presence of 5 μg/ml PcrV, PopB, or PBS, in plates coated with antibodies against IL-17A for a color assay as per manufacturer’s specifications (ImmunoSpot). The IL-17A secreting cells were quantified using a CTL immunospot reader. Biological negative controls were maintained as PBS mice group while technical negative controls were cells without any protein treatment.

### Cytokine Determinations

Lung cells were incubated with 10 μg/ml PcrV, PopB, or PBS for 48 h at 37°C. Supernatants were collected and analyzed with U-PLEX kits for cytokines: IFN-γ, IL-2, IL-6, IL-17A, and TNF-α. Cytokine concentrations were determined using an MSD plate reader with associated analytical software (Meso Scale Discovery, Rockville, MD). For post-challenge cytokine determination, cells were not stimulated with PcrV or PopB. Instead, mice lungs were assessed to determine *in vivo* pro-inflammation resulting from bacterial challenge. Control groups were maintained as described above.

### *Pseudomonas Aeruginosa* Challenge

The mucoid Pa strain mPA08-31 was streaked onto *Pseudomonas* isolation agar (PIA) and incubated overnight at 37°C with shaking at 180 rpm. A 200 µl aliquot from the overnight culture was inoculated into 20 ml of LB and grown at 37°C with 250 rpm shaking the A_600 nm_ reached ∼0.3. Pa were collected by centrifugation, washed once, resuspended in PBS, and diluted to 4x10^7^ CFU/30 µl. On day 56, mice were anesthetized by isoflurane and challenged IN. Clinical body scores have been documented as follows: 1: Bright, alert, reactive, shiny hair coat, no piloerection; 2: Quiet, alert, reactive, early-stage piloerection, mild dehydration; 3: Quiet, not very reactive, mild piloerection, moderate dehydration; 4: Minimally reactive, severe piloerection, dull, dirty hair, hunched posture, respiratory distress. On day 3 post-infection, mice (n = 4) from each group were euthanized and the lungs were collected and processed to assess the immune response in terms of secreted cytokine levels. Additionally, the CFU/lung was enumerated for each mouse by plating a portion of the lung extract on PIA.

### Correlation Studies

Linear correlation studies were carried out using bivariate correlation in the form of Pearson r. They were further analyzed via simple linear regression. Both operations were carried out with 95% confidence interval, using GraphPad Prism 8.1.2.

1) Cytokines and lung burden. Post-challenge lung cytokines and lung burden were checked for linear correlation as described above.
2) OPK and lung burden *in vitro* bacterial killing ability of the serum was checked with *in vivo* lung burden to find out a possible correlation between them.

### Statistical Analysis

GraphPad Prism 8.1.2 was used to generate graphics and to perform all statistical comparisons. Differences among treatment groups were analyzed using two-way ANOVA. Challenge groups were compared to PBS with Dunnett’s multiple comparisons tests. A *p* value of less than 0.05 was considered significant for all comparisons. *p < 0.05; **p < 0.01; ***p < 0.001. Pearson’s r values were mentioned at appropriate places along with R squared values from simple linear regression analyses.

## Results and Discussion

### L-PaF Interacts With ME

Having previously demonstrated broad protection of L-PaF, we sought to produce two multimeric formulations that were unique in size, composition, and induction of the immune system *via* different mechanisms. The first formulation examined was an o/w emulsion referred to as ME (MedImmune Emulsion). ME is ∼100 nm in size and can thus be taken up directly by dendritic cells (Marasini et al., 2017; Corthesy and Bioley, 2018). We determined that L-PaF was associated with ME by measuring the particle size distribution and zeta potential of ME before and after mixing with L-PaF. The zeta potential of ME mixed with L-PaF had a slight positive value as compared to the negative values of ME or L-PaF alone, which suggests that their association significantly alters the resulting particle’s surface features (Table 1). With respect to particle size, L-PaF had a high polydispersity index (PdI >80%) with a size distribution ranging from about 8 to 400 nm, suggesting that L-PaF has the tendency to form aggregates (Table 1). Conversely, ME had a unimodal size distribution with a low polydispersity index (PdI <10%) (Table 1). Mixing L-PaF with ME increased the particle size from 113 to 143 nm without an increase in polydispersity (PdI <10%) (Table 1). In other work, it was found that the addition of BECC438 did not significantly change the size of the ME (data not shown).

**TABLE 1:** Summary table of particle characteristics of L-PaF, ME, and L-PaF + ME.

Name	Zeta potential (mV)	Z_average (d.nm.)	Polydisperity
ME	−23.4 ± 1.6	113.3 ± 0.6	0.091 ± 0.016
L-PaF	−6.31 ± 0.23	35.9 ± 4.1	0.835 ± 0.057
ME + L-PaF	1.47 ± 0.8	144.6 ± 2.9	0.081 ± 0.013

**FIGURE 1:**
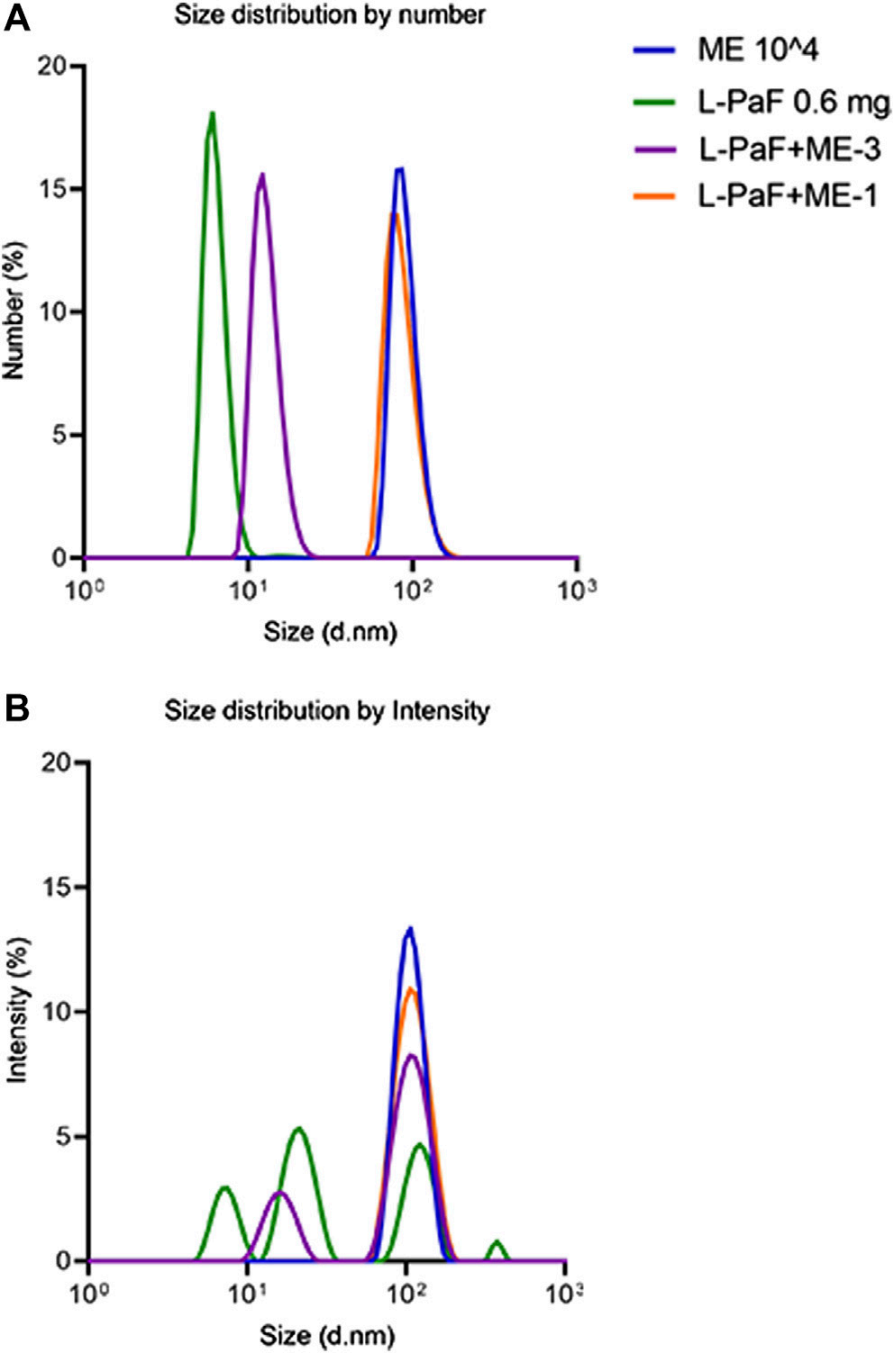
Particle size determined by multi-angle dynamic light scattering (MADLS). **(A)** Number weighted size distribution of 0.6 mg/mL L-PaF alone (green), 10,000-fold diluted ME emulsion alone (ME, blue plot), L-PaF + 10,000X diluted ME (ME-3) and L-PaF + 1,000X diluted ME (ME-1, orange). **(B)** Intensity weighted size distribution of 0.6 mg/mL L-PaF alone (green), 10,000X diluted ME emulsion (blue), L-PaF + 10,000X diluted ME (purple) and L-PaF + 1,000X diluted ME (orange).

To investigate the change of particle size with the addition of L-PaF to ME further, we performed MADLS measurements (Figure 1) (Naiim et al., 2015; Austin et al., 2020). These results confirmed that L-PaF is heterogeneous with four distinct particle size populations based on intensity-based measurements, however particle numbers indicated that the smallest species (∼8 nm) was dominant. Meanwhile, ME is largely homogenous in size with an ∼100 nm particle size. When 10,000X diluted ME (designated ME-3) was mixed with L-PaF, there were two populations of particle size based on intensity (Figure 1A). One population was ∼10 nm close to the size of L-PaF alone and the other was ∼100 nm, suggesting not all L-PaF was incorporated into ME particle (Figure 1). In contrast, when 1,000X diluted ME was mixed with L-PaF (designated ME-1), only one population of particle size was observed for both based on number and intensity (Figure 1B). Our results confirmed that the mixing of L-PaF with ME leads to the incorporation of L-PaF into the ME particle with an excess of ME leading to a single homogenous population L-PaF/ME nanoparticles.

**FIGURE 2:**
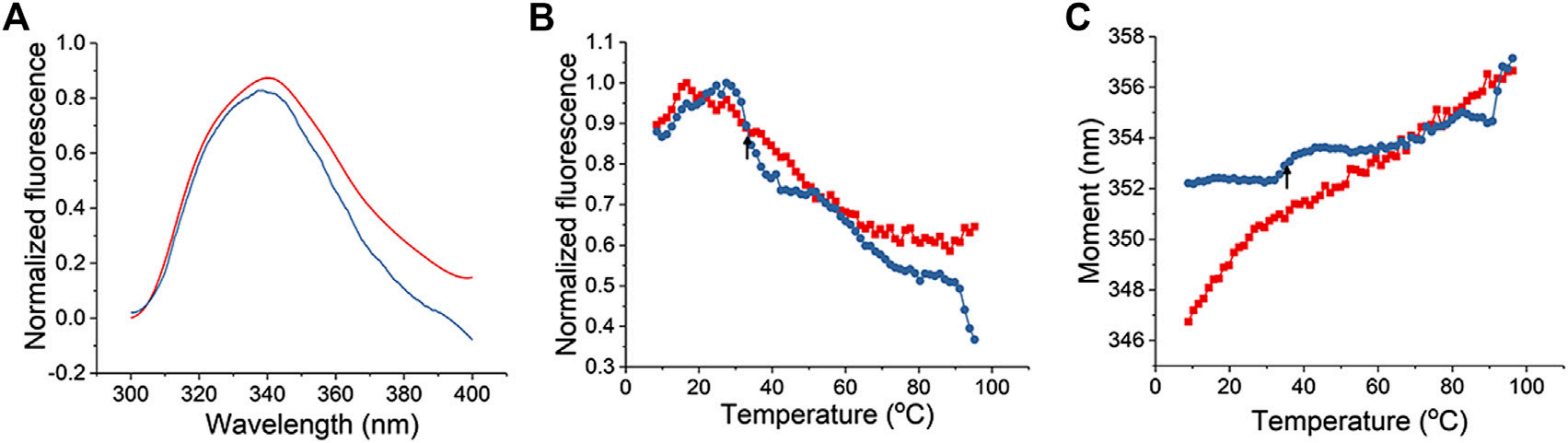
Tertiary structure changes in L-PaF monitored in the presence and absence of ME using intrinsic fluorescence spectroscopy. **(A)** Normalized fluorescence spectra of L-PaF and L-PaF with ME at 10°C. Thermal unfolding profiles were monitored by **(B)** fluorescence intensity and **(C)** MSM peak position. The plots for L-PaF and L-PaF + ME are in red and blue, respectively. The arrow indicates the thermal transition observed for L-PaF when associated with the ME.

As a second method to assess the L-PaF interaction with ME, we monitored intrinsic (tryptophan) fluorescence of the L-PaF. The intrinsic fluorescence spectra of L-PaF in PBS with (blue) and without (red) ME at 10°C were obtained and normalized to allow direct comparison of formulations (Figure 2). PBS and ME alone scans were also obtained so that these background measurements could be subtracted to obtain only the spectral data of the L-PaF. ME by itself showed an unexpected fluorescence signal from 300 to 400 nm when exited at 295 nm, thereby necessitating its subtraction. Whether with or without ME, L-PaF exhibited similar emission spectra with maximal emission around 339 nm at 10°C. This confirmed that the overall tertiary structure of L-PaF was not changed significantly by its association with ME (Figure 2A). Thermal melting profiles using the normalized fluorescence intensities and moment (MSM peak position) were plotted to compare the thermal transitions for both L-PaF alone and L-PaF with ME (Figures 2B,C). While neither plot of the thermal melts for L-PaF demonstrated an obvious transition (red plots), the L-PaF associated with ME showed a noticeable thermal transition at about 36°C (indicated by black arrow for each blue plot).

### L-PaF Interacts With Mucus-Interacting Chi-C48/80 (Chi)

As a second unique particulate formulation, L-PaF was bound to chitosan (∼600 nm particles). Chitosan nanoparticles are somewhat unusual due to their positive charge which allows them to interact with negatively charged polysaccharides such as mucin, which is the major component of mucus layer on the surface of nasal epithelial cells. The DLS and zeta potential measurement confirmed the interaction of our preparation of Chi with mucin (Table 2). The Z-average value (size) of Chi increased from 635 to 709 nm after adding mucin while the zeta potential was decreased from 17.2 mV to −1.0 mV for Chi-mucin (Table 2). To determine the percentage of L-PaF associated the Chi, we assessed protein adsorption efficiency to Chi by measuring the soluble L-PaF after centrifugation of the binding reaction and found that approximately 35% of the L-PaF was associated with Chi. The relatively low adsorption efficiency may be due to the pI of L-PaF (pI = 6.1). We were unable to determine the amount of BECC438 bound to Chi, however, based on the interaction of Chi with mucin, we assume that the BECC438, a negatively charged molecule, is able to interact with the Chi.

**TABLE 2:** Summary table of particle characteristics of Chi, mucin, and Chi + mucin.

Name	Zeta potential (mV)	Z_average (d.nm.)	Polydisperity
Chitosan-C48/80 (Chi)	17.2 ± 0.36	635.40 ± 8.66	0.228 ± 0.050
Mucin	−21.7 ± 0.49	370 ± 43.26	0.508 ± 0.080
Chitosan-C48/80 + mucin (Chi-mucin)	−1.0 ± 0.43	709 ± 11	0.240 ± 0.012

### L-PaF is Immunogenic Regardless of Formulation

**FIGURE 3:**
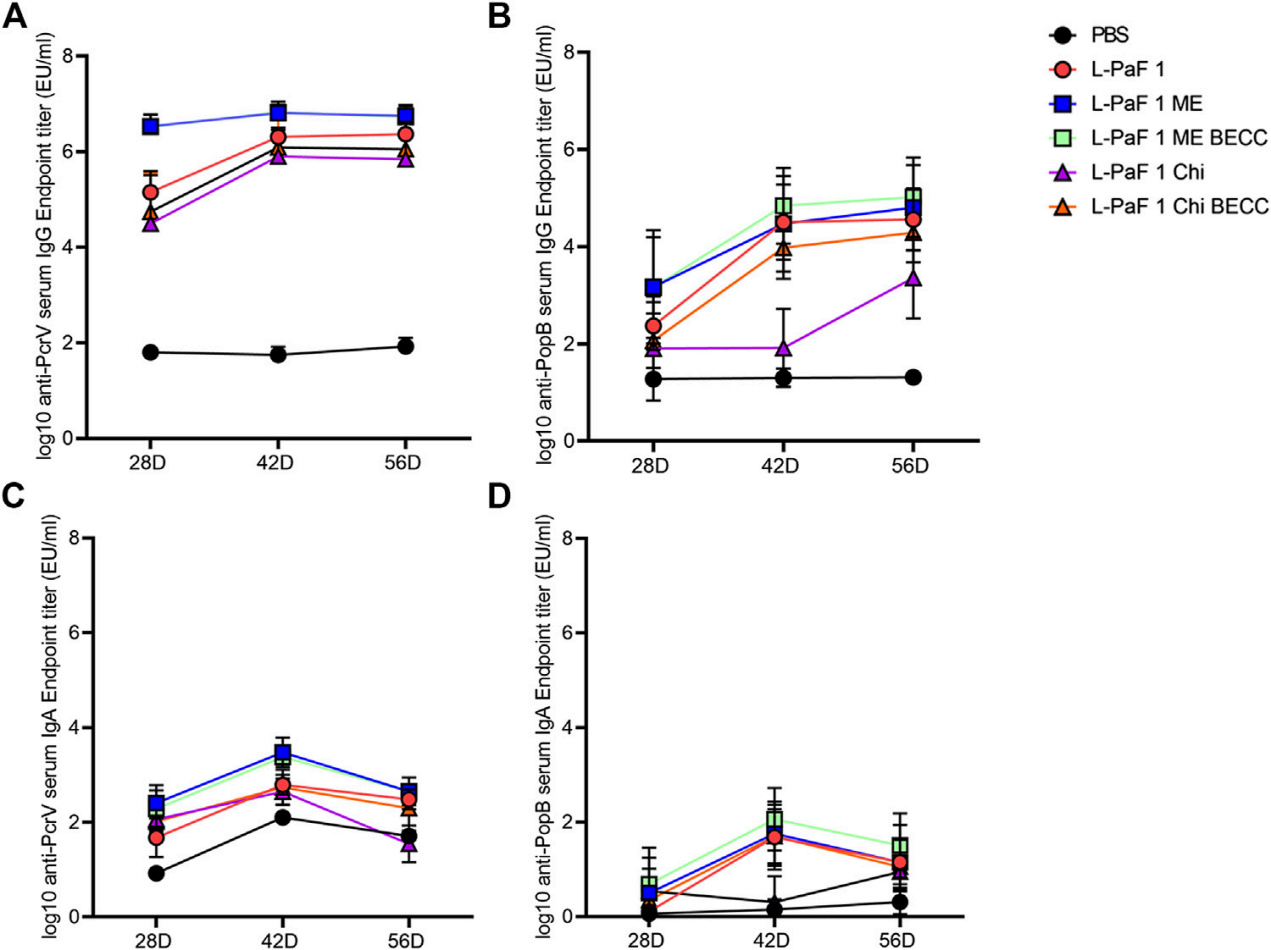
Kinetics of serum IgG and IgA. Mice were vaccinated IN a prime-boost manner with the primary dose on day 0, followed by two booster doses 14 days apart from each other. Blood samples were assessed for the presence of anti-PcrV IgG **(A)**, IgA **(C)** and anti-PopB IgG **(B)**, IgA **(D)** by ELISA. Individual titers were represented as EU/ml. Each point represents the mean while error bars represent the SD of each group (n = 10 mice/group).

Mice were vaccinated using a prime-boost-boost scheme at 14-days intervals. We found that all formulations containing L-PaF induced comparable amounts of serum antibodies (Figure 3). While L-PaF BECC438/ME elicited the highest antibody responses among all the groups, no statistical significance was detected in most cases (Figure 3). In contrast, L-PaF Chi at day 42 did not induce comparable antibody levels (for both IgG and IgA) against PopB, while an increase was observed by day 56 (Figures 3B,D). A general trend of increasing antibody production was observed for IgG with the lowest titers at day 28 and similar titers seen at days 42 and 56 against both proteins (Figures 3A,B). In contrast, the highest IgA titers were observed at day 42 with a decline at day 56 (Figure 3C,D). Significant class switching was observed mainly for L-PaF BECC/ME group.

**FIGURE 4:**
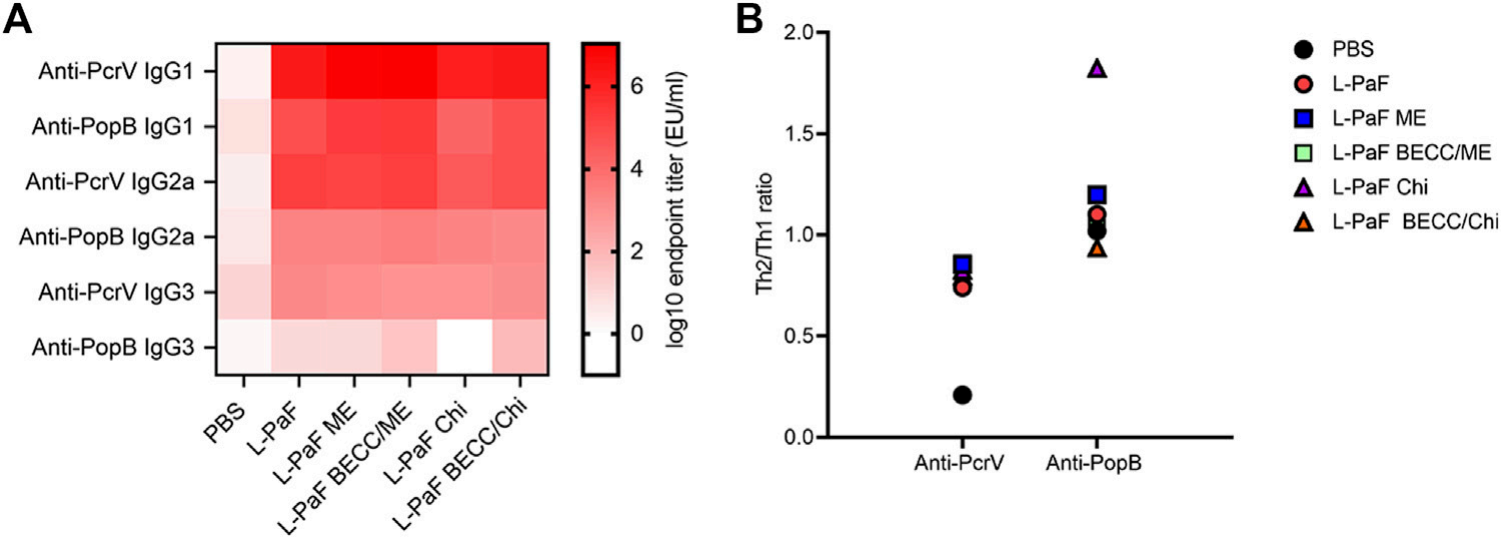
Heat-map of serum IgG subtype antibody titers. Sera from IN vaccinated mice were assessed for the presence of different IgG subtypes on day 56 post immunization **(A)**. Th2/Th1 ratio was assessed as a measure of Th bias **(B)**. Titers were plotted as EU/ml for the heat map. Each dot in the second panel represents a mean value of [IgG1/(IgG2a + IgG3)] from EU/ml values of a pooled ELISA data.

Measurement of different IgG subtypes in day 56 sera (Figure 4A) showed a slight Th1 bias against PcrV and Th2 bias against PopB (Figure 4B). Heat map was generated where the *X* axis is showing the groups and *Y* axis is showing the antibodies tested. A reference bar has been added indicating antibody titers (EU/ml). The most abundant subtype was found to be IgG1 followed by IgG2a and IgG3. A clear difference was observed in the cases of IgG2a and IgG3, where PcrV caused a greater induction of these antibodies than PopB. The literature suggests that IgG2a and IgG3 (Th1-specific) can fix complement better than IgG1 (Th2-specific). IgG3 can also bind directly to bacteria to provide protection is the same way that IgG1 does (Lefeber et al., 2003). This might have been the cause of the higher OPK response seen previously for PopB vaccinated mice in the absence of complement *in vitro* (using heat-inactivated serum) (Das et al., 2020). Conversely, PcrV appears to impart its potency *in vivo* by activating the host immune system in a way that leads to complement mediated lysis of Pa. Activation of the adaptive immune response following L-PaF vaccination, thus provides early hints about its immunogenicity and efficacy as a Pa vaccine.

### L-PaF Vaccination Leads to a Maintained Th1-Th17 Skewed Cytokine Profile in the Lungs of Immunized Mice Prior to Challenge

**FIGURE 5:**
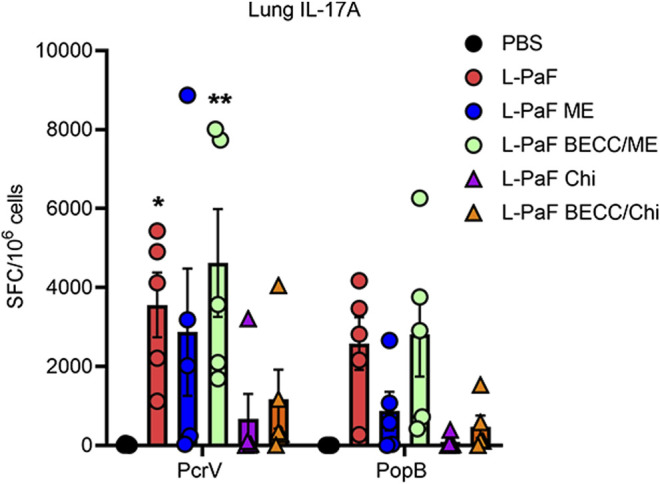
Lung cells secretes IL-17A following antigen-specific *ex vivo* stimulation. Lung cell suspensions were prepared as described in the text. They were treated with 5 μg/ml of either PcrV or PopB and incubated for 24 h at 37°C. IL-17A secreting cells were enumerated by ELISpot and were plotted as Spots Forming Cells (SFC)/10^6^ cells. Data were plotted as actual values from individuals ± SD (n = 5) in each group. Statistical significance was calculated by comparing the PBS group with their immunized counterparts using two-way ANOVA (Dunnett’s multiple comparison test). *p < 0.05, **p < 0.01.

While B cell activation was found to be Th1 skewed, T cell activation followed a Th1-Th17 skewing following vaccination. On day 56, extracted lungs from vaccinated mice (n = 5) were processed and IL-17A secreting cells were determined (Figure 5). Cells from mice vaccinated with L-PaF alone or with BECC438/ME that were treated with PcrV had significantly higher frequencies of IL-17A secreting cells compared to the control groups. While IL-17A secreting cells were also found after treatment with PopB, no statistical significance was observed. Negative control cells (not receiving any protein stimulation) did not show any significant IL-17A secreting cells either (data not shown).

**FIGURE 6:**
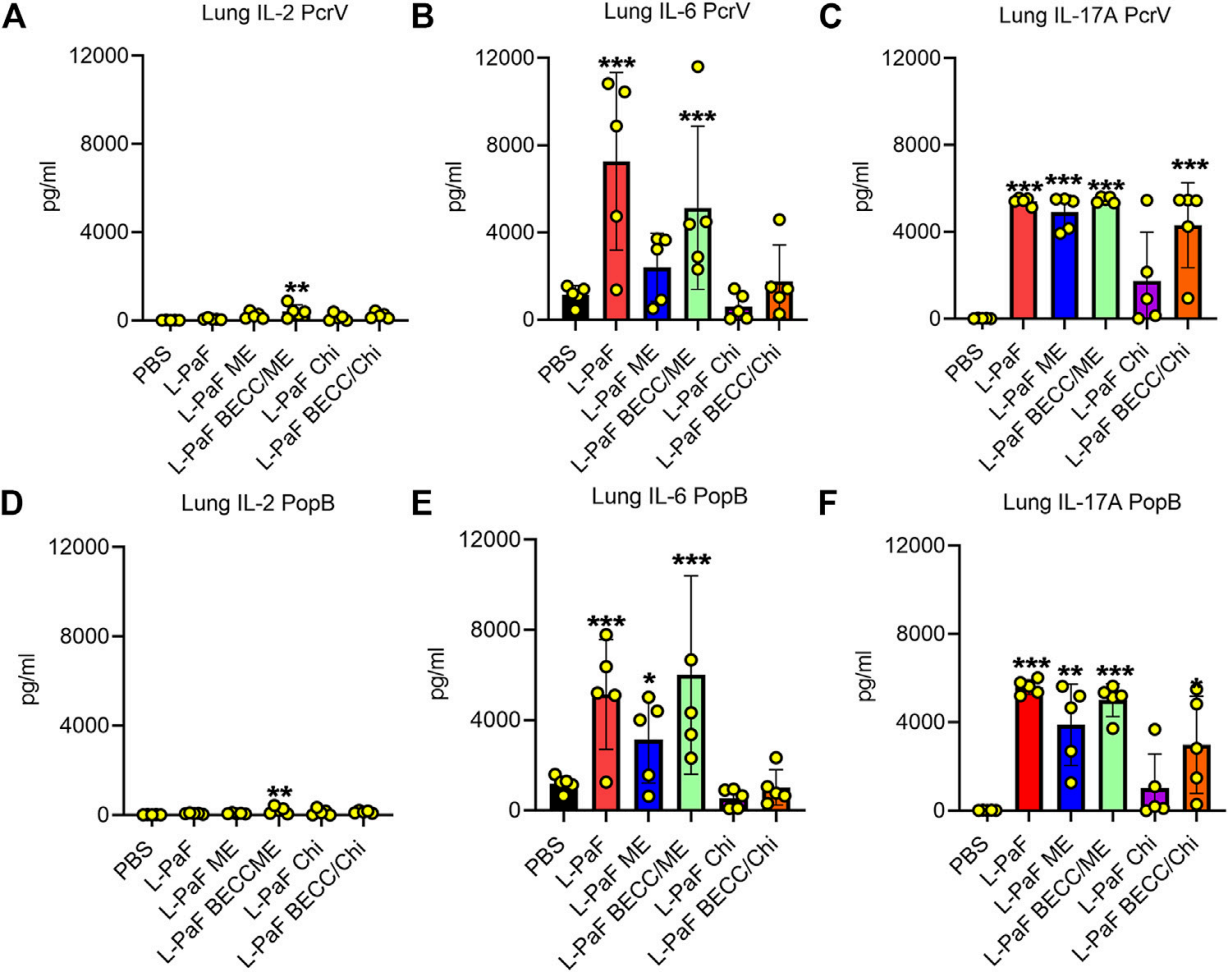
Lung cells secretes pro-inflammatory cytokines following stimulation with PcrV or PopB. Lung cell suspensions were prepared and single cell suspensions were treated with 10 μg/ml of either PcrV or PopB and incubated for 48 h at 37°C. Secretion of IL-2 **(A, D)**, IL-6 **(B, E)** and IL-17A **(C, F)** were noted as a response of either PcrV or PopB stimulation. Amounts of cytokines were determined by MesoScale Discovery (MSD) analysis as per manufacturer’s instructions and were presented as pg/ml/10^6^ cells. Data were plotted as actual values from individuals ± SD (n = 5) in each group. Statistical significance was calculated by comparing the PBS group with their immunized counterparts using two-way ANOVA (Dunnett’s multiple comparison test). *p < 0.05, **p < 0.01, ***p < 0.001.

We also used pre-challenge lung cells to assess the secretion of different cytokines after stimulation with PcrV or PopB. Th1-Th17 bias was measured based on detection of potent Th1 and Th17 cytokines, namely, IFN-γ, the master regulator IL-2, pro-inflammatory IL-6, TNF-α, and IL-17A. Although no statistical significance was found for IFN-γ and TNF-α (Supplementary Figure S2), the other three cytokines showed different secretion patterns among the groups. (Figure 6). IL-2, IL-6, and IL-17A were significantly upregulated in lung cells from the L-PaF BECC438/ME immunized mice (after stimulation with either PcrV or PopB). IL-2 is the master regulator that activates various downstream pathways leading to immune response. Significant upregulation was seen for PcrV and PopB treated cells collected from L-PaF BECC438/ME immunized mice (Figure 6A,D). PcrV and PopB treated cells collected from L-PaF and L-PaF BECC438/ME immunized mice, respectively, also had elevated amounts of IL-6 (Figure 6B,E). Conversely, significant secretion of IL-17A was detected from cells from all the immunized mice groups except for L-PaF Chi (Figure 6C,F). Cells from mice vaccinated with PBS had very low or no response under any treatment conditions. Negative control group, where no protein stimulation was provided, only had significant IL-17A in L-PaF BECC438/ME immunized lung cells (<500 pg/ml) (data not shown), but the quantity was much lower than that seen for the treated groups (>3,000 pg/ml). The ability to generate IL-17A without any treatment indicates the immunogen’s potency towards protection. Literature suggests the positive association of IL-17A with mucosal vaccination in the form of memory B cell generation (Oppmann et al., 2000; Khader et al., 2009). Generation of IL-2 and IL-6 has their own merits too. Post-immunization moderate response of these cytokines ensures the generation of a protective immune response (Tanaka et al., 2014). Moreover, IL-6 helps the host immune system in the transition of innate to acquired (Jones 2005).

### Protective Efficacy

#### The *in Vivo* Lung Burden was Reduced Significantly for the ME-and ME BECC438-Containing Groups

**FIGURE 7:**
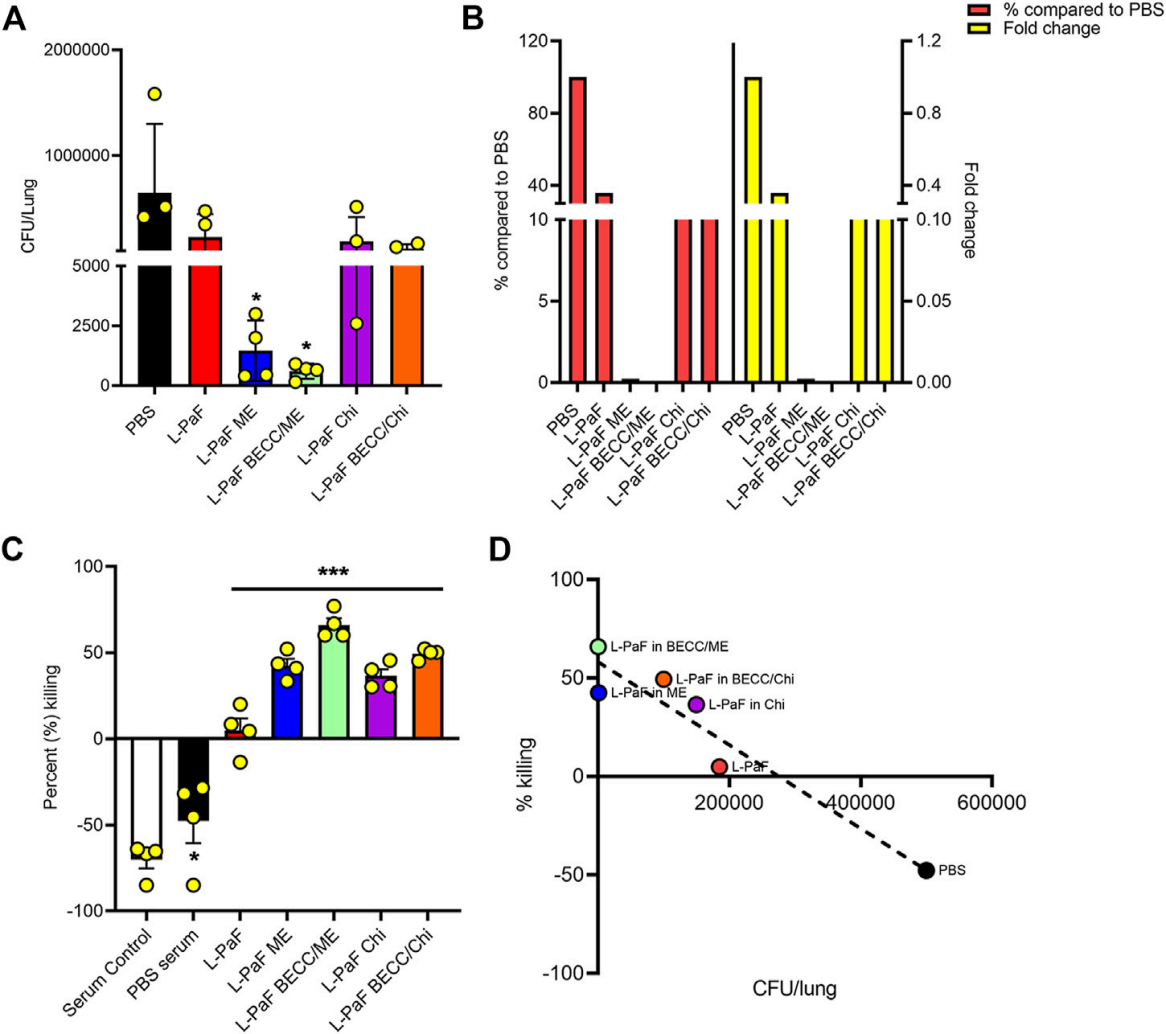
*In vivo* and *in vitro* protective efficacy studies. For *in vivo* efficacy studies, on day 56 post immunization, mice were challenged IN with mucoid Pa strain mPa08-31 at a concentration of 4x10^7^ CFU/30µl/mouse and CFU/lung were determined on three DPI (Days Post Infection) (n = 4) **(A)**. The relative lung burdens were compared to PBS and the fold-change was then calculated **(B)**. For *in vitro* efficacy studies, an OPK assay was carried out as described in the text. Briefly, the murine macrophage cell line J774A.1 was combined with mPa08-31at an MOI of 0.1. Heat-inactivated serum was mixed at a ratio of 1:500. OPK activity was determined at 30min (n = 4) **(C)**, which was then checked for any correlation with *in vivo* lung burden **(D)**. OPK was determined using the following formula: (T_0_-T_30_)/T_0_x100. T_0_ and T_30_ were CFUs at time 0 and 30min, respectively. Each dot in **(A)** and **(C)** represents individual values from either a mouse lung or OPK sample mix. Statistical significance for **(A)** and **(C)** were calculated as previously explained in Figures 5, 6. Pearson’s r coefficient was determined for **(D)** to allow assessment of the correlation (r = −0.9538). Simple linear regression was carried out with 95% confidence level (R squared = 0.9098, deviation from zero = significant). *p < 0.05, ***p < 0.001.

On day 56, we challenged the vaccinated mice with the clinical mucoid isolate, Pa mPa08-31 and monitored their morbidity over a 3-day period. No mortality was seen during this time. While PBS mice were sick with higher health scores, indicating morbidity, the immunized mice displayed little or no morbidity. Extracted lungs were processed and colony forming units (CFU) lung burden was determined by dilution plating of the homogenates on *Pseudomonas* Isolation Agar (PIA) (Figure 7A). Lungs from L-PaF ME and L-PaF BECC438/ME vaccinated mice had significantly lower bacterial burden than the other groups. Lung bacterial burden is a clear indicator of the immunogen’s protective efficacy in mice, with the highest fold change (and % compared to PBS) observed for the L-PaF BECC438/ME vaccinated group (Figure 7B). We have previously demonstrated that L-PaF is protective against multiple Pa serotypes (Das et al., 2020), so here we focused on showing that the protective efficacy of L-PaF could be improved by formulating it into a nanoparticle containing the TLR4 agonist BECC438.

### Serum From the Immunized Groups Reduced Bacterial Burden *in Vitro*

Opsonophagocytic killing (OPK) is an important marker of *in vitro* functional protective efficacy (Pier et al., 1987; Yang et al., 2017; Das et al., 2020). Sera from mice immunized with all L-PaF formulations were found to possess significant bactericidal capability (Figure 7C). Serum from immunized groups showed a bacterial killing ability in the range of 4–76%, with highest killing potential seen for sera from mice vaccinated with L-PaF BECC438/ME. Although L-PaF BECC438/Chi group showed a slightly higher killing ability than L-PaF ME *in vitro*, this was at odds with protective capacity *in vivo*. A negative percent killing indicates into bacterial replication and this was only seen for the serum control and PBS groups. Although L-PaF alone serum did not possess a striking level of OPK ability, it was significantly better that the controls in which the Pa population expanded. When the *in vivo* and *in vitro* data were compared, a clear negative correlation was found, meaning that a higher *in vitro* killing (OPK) coincides with a reduced bacterial burden in the lung (Figure 7D). These results demonstrate the immunogenicity and protective efficacy of L-PaF with or without formulation.

### Colonization Reduction and Lesser Morbidity Associates With Post-challenge *in Vivo* High IL-17A and Low TNF-α

**FIGURE 8:**
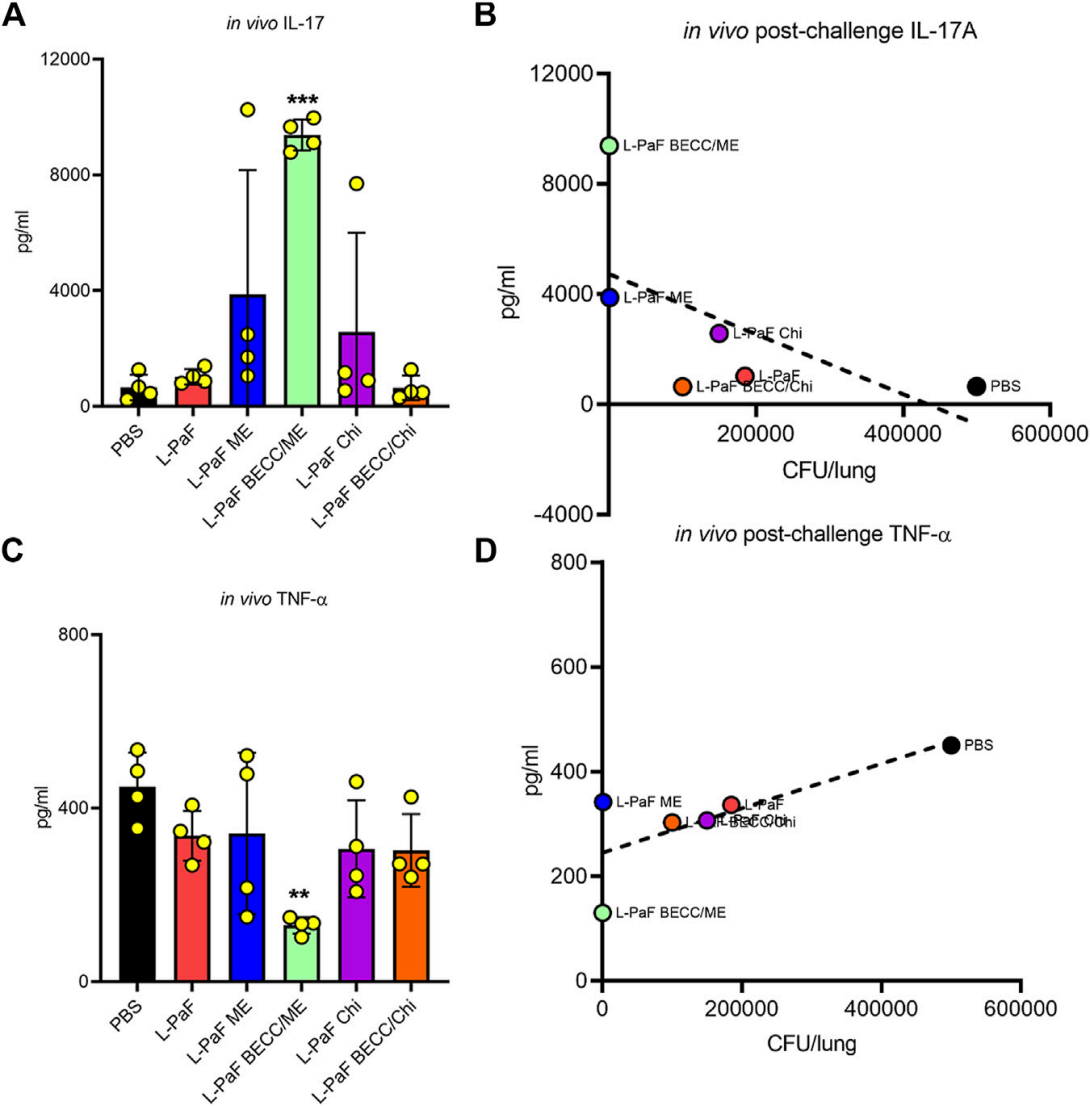
The *in vivo* post-challenge cytokines were assessed to provide a potential immune correlate of protection. The effects of post-challenge cytokines were measured in mice lung infected with the Pa isolate mPa08-31. Lung cell suspensions were left untreated for 48 h at 37°C. IL-17A **(A)** and TNF-α **(C)** were measured and plotted as actual values from individuals ± SD (n = 4) in each group. Correlation of *in vivo* IL-17A **(B)** and *in vivo* TNF-α **(D)** were measured as described previously. Pearson’s r for these two graphs were −0.5977 and 0.7565, respectively. Simple linear regressions were carried out, where R squared values were 0.3573 and 0.5722, respectively. **p < 0.01, ***p < 0.001.

To begin dissecting the mechanism of protection elicited by the best L-PaF formulation(s), we further assessed lung cells from the challenged mice to detect secretion of pro-inflammatory cytokines, IL-17A and TNF-α, which play a crucial role following immunization and challenge (Figure 8). Unlike the pre-challenge cells, these cells were not stimulated with PcrV or PopB prior to cytokine measurement. Thus, cytokines here were generated because of the infection/challenge regardless of whether the mice were vaccinated. The L-PaF BECC438/ME group showed significantly higher levels of IL-17A secretion than the other groups (Figure 8A). This was inversely correlated with lung burden: i.e., less IL-17A produced post-challenge equates to a higher lung burden (Figure 8B). This same group had significantly less TNF-α in comparison to the other groups (Figure 8C). The literature suggests that IL-17A is an important cytokine for proper immune stimulation and maintenance against Pa infection (Das et al., 2020). Similar trend was observed in the present study with high IL-17A present in pre-challenge lung cells (Figures 5, 6). Furthermore, another trend that was observed here was that a sustained presence of pro-inflammatory cytokines (IL-2, IL-6, and IL-17A) prior to infection deemed important to reduce the extent of the infection. TNF-α concentrations were maintained low during this period. On the other hand, a high TNF-α following Pa challenge was positively associated with high bacterial burden (Figure 8D). As already described, difference in expression of pre-challenge TNF-α was not statistically significant among the groups. We hypothesize this high amount of TNF-α in the groups except for L-PaF BECC438/ME, disrupts the host immune homeostasis. It can thus be concluded that high post-challenge *in vivo* IL-17A acted as a protective barrier against infection just like the pre-challenge levels, while a high TNF-α following infection was positively associated with lung burden.

## Conclusion

Our previous work has demonstrated the protective efficacy of the L-PaF against a homologous and heterologous Pa challenge. However, in humans monomeric antigens tend to not elicit a strong protective response and require a multimeric presentation (Marasini et al., 2017; Corthesy and Bioley, 2018). As such this proof-of-concept study sought to examine the efficacy of L-PaF in two multimeric formulations with unique sizes, compositions, and mechanism of induction of the immune system. ME is a w/o emulsion with a diameter of ∼100 nm which should be taken up by dendritic cells. In contrast, the chitosan formulation is ∼600 nm in diameter and binds to mucin to potentially act with a depot effect. We found that, while both formulations provide some protection, the ME formulation is superior to the chitosan formulation. The further contribution of the TLR4 agonist, BECC438, was also monitored. Comparative analysis of these newly formulated immunogens showed that the L-PaF BECC438/ME formulation was highly immunogenic and provided the best protective efficacy. While we see high antibodies titers, these antibodies do not provide complete protection as we have seen with our similar vaccine against *Shigella* spp. and *Salmonella enterica* (Martinez-Becerra et al., 2012; Martinez-Becerra et al., 2013a; Martinez-Becerra et al., 2013b; Martinez-Becerra et al., 2018). In concordance with these findings, the observed Th1/Th17 skewed immune response resulted in upregulation of different pro-inflammatory cytokines pre-challenge, further contributing to protection. On the other hand, the presence of elevated TNF-α post-challenge was found to directly correlate with higher bacterial burden in mice lung. Because of the nature of the L-PaF BECC438/ME emulsion (size, composition, and surface biophysical properties), it is possible that this novel formulation is better suited for use in humans than is the L-PaF alone.

## Data Availability

The original contributions presented in the study are included in the article/Supplementary Material, further inquiries can be directed to the corresponding author.
