# Supplementary material for: Effect of Two Unique Nanoparticle Formulations on the Efficacy of a Broadly Protective Vaccine Against Pseudomonas Aeruginosa
Source: Front Pharmacol. 2021 Aug 18;12:706157. doi: 10.3389/fphar.2021.706157 (PMC8416447; doi:10.3389/fphar.2021.706157)
Supplement: Supplementary file 1 [file DataSheet1.docx]

Supplemental Information

**Effect of two unique nanoparticle formulations on the efficacy of a broadly protective vaccine against *Pseudomonas aeruginosa***

Debaki R Howlader^1^, Sayan Das^1^, Ti Lu^1^, Gang Hu^1^, David J Varisco^2^, Zackary K Dietz^1^, Sierra P Walton^1^, Siva Sai Kumar Ratnakaram^1^, Francesca M Gardner^2^, Robert K Ernst^2^, William D Picking^1^, and Wendy L Picking^1*^

^1^Department of Pharmaceutical Chemistry, University of Kansas, Lawrence, Kansas 66047 and ^2^Department of Microbial Pathogenesis, University of Maryland, Baltimore, MD 21201

*Corresponding Author:

Wendy L Picking, [wendy.picking@ku.edu](mailto:wendy.picking@ku.edu)

Keywords: *Pseudomonas*, T3SS vaccine, L-PaF, Nanoparticle vaccine, Protein formulations, Opsonophagocytosis, IL-17.

Supplementary Figure S1.

**
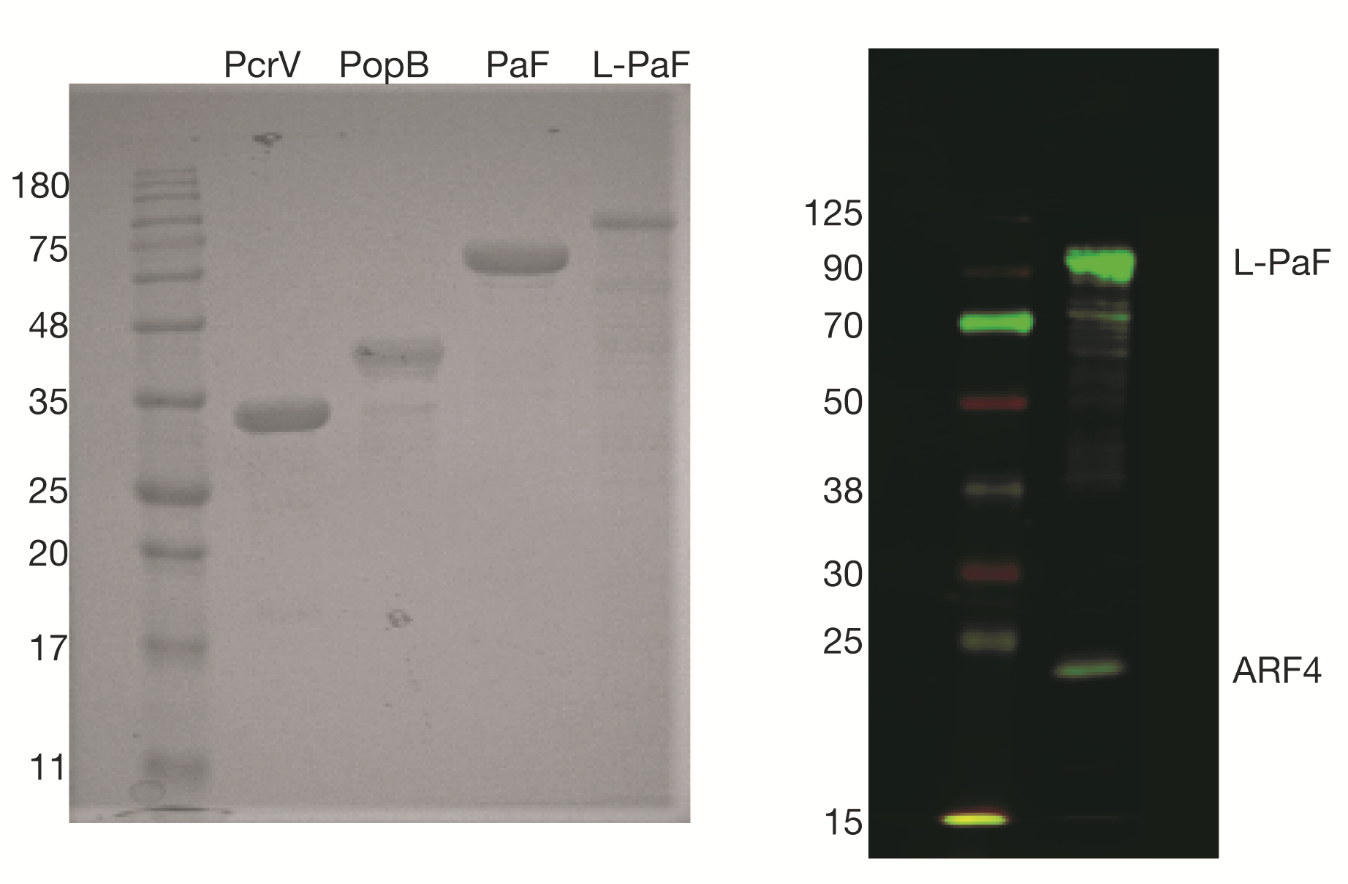
**

**Supplementary Figure S1. Preparation of the recombinant proteins used in this study.** Four recombinant proteins were tested in this study. PcrV (tip protein) and PopB (first translocator protein) from the *Pseudomonas aeruginosa* T3SS were purified as described. Then two novel proteins were prepared in which PcrV was fused with PopB (to give PaF) and in which PaF was fused with LTA1 (L-PaF). These four proteins are shown after separation using SDS-PAGE (0.05 µg) and staining with One-Step Blue protein gel stain (Biotium).

This figure was originally presented in Das S, Howlader DR, Zheng Q, Ratnakaram SSK, Whittier SK, Lu T, Keith JD, Picking WD, Birket SE, Picking WL. Development of a Broadly Protective, Self-Adjuvanting Subunit Vaccine to Prevent Infections by *Pseudomonas aeruginosa*. Front Immunol. 2020 Nov 17;11:583008. doi: 10.3389/fimmu.2020.583008. PMID: 33281815; PMCID: PMC7705240.

Supplementary Figure S2.

**(A)**

**(B)**

**(C)**

**(D)**

**Supplementary Figure S2. Cytokine secretion from lung cells.** Lung cells secretes pro-inflammatory cytokines following stimulation with PcrV or PopB. Lung cell suspensions were prepared and single cell suspensions were treated with 10 µg/ml of either PcrV or PopB and incubated for 48 h at 37°C. Secretion of IFN-γ **(A, C)** and TNF-α **(B, D)** were noted as a response of either PcrV or PopB stimulation. Amounts of cytokines were determined by MesoScale Discovery (MSD) analysis as per manufacturer’s instructions and were presented as pg/ml/10^6^ cells. Data were plotted as actual values from individuals ± SD (n = 5) in each group. Statistical significance was calculated by comparing the PBS group with their immunized counterparts using two-way ANOVA (Dunnett’s multiple comparison test). ns – non-significant.
